# Grape seed proanthocyanidin extract protects from cisplatin-induced nephrotoxicity by inhibiting endoplasmic reticulum stress-induced apoptosis

**DOI:** 10.3892/mmr.2014.1883

**Published:** 2014-01-03

**Authors:** ZHAOLI GAO, GUANGYI LIU, ZHAO HU, XING LI, XIANGDONG YANG, BEI JIANG, XIANHUA LI

**Affiliations:** 1 Department of Nephrology, Qi Lu Hospital of Shandong University, Jinan, Shandong 250012, P.R. China; 2 Ministry of Education Key Laboratory of Experimental Teratology and Institute of Molecular Medicine and Genetics, Shandong University School of Medicine, Jinan, Shandong 250012, P.R. China

**Keywords:** apoptosis, cisplatin, endoplasmic reticulum stress, grape seed proanthocyanidin extract, nephrotoxicity

## Abstract

Cisplatin (CP) is used as an antineoplastic drug in the clinic, but its nephrotoxicity limits its use. Grape seed proanthocyanidin extract (GSPE) is a powerful antioxidant. In this study, we investigated whether GSPE can prevent CP-induced nephrotoxicity and explored the underlying mechanism. Male C57/BL6 mice were randomly divided into four groups: control group (N), CP group (C), receiving an intraperitoneal (ip) injection of 20 mg/kg CP, GSPE group (G), receiving an intragastric (ig) dose of 500 mg/kg GSPE, and CP+GSPE group (C+G), where ig administration of GSPE was performed 30 min prior to ip injection of CP, followed by an additional ig administration of GSPE 72 h later. Blood and kidney samples were collected 120 h after treatment. The pathological changes in the kidney were examined by periodic acid-Schiff (PAS) staining, while the protein levels of glucose-regulated protein 78 (GRP78), phosphorylated-extracellular signal-regulated kinase (p-ERK) and caspase-12 were examined by western blotting and immunohistochemical staining. Apoptosis was examined by a terminal deoxynucleotidyl transferase dUTP nick-end labeling (TUNEL) assay. Compared to the CP group, the CP+GSPE group had a significant decrease in the level of blood urea nitrogen (BUN), serum creatinine (Scr) and reduced renal index (RI) (P<0.05), and showed limited histopathological damage. The number of TUNEL-positive cells was significantly reduced in the CP+GSPE group compared to the CP group (P<0.05), and the protein expression of GRP78, p-ERK and caspase-12 was significantly reduced in the CP+GSPE group (P<0.05). We conclude that GSPE can protect the renal function from CP-induced nephrotoxicity and can attenuate the endoplasmic reticulum (ER) stress-induced apoptosis via regulation of the caspase-12 pathway.

## Introduction

Cisplatin (CP) or *cis*-Diamminedichloroplatinum (II)*,* is widely used as an antitumor agent for the treatment of testis, bladder, lung and ovarian cancer. However, severe side-effects such as acute kidney injury (AKI), gastrointestinal toxicity, and ototoxicity limit its use in the clinic. In particular, AKI is a major side-effect (1–4). CP is toxic to the renal proximal tubules (1,5,6). The nephrotoxic potential of CP is multifactorial, with the major factor being the induction of tubular cell apoptosis. The induction of tubular cell apoptosis by CP has been intensively investigated in the past decades. A number of apoptotic pathways have been examined, including the intrinsic, extrinsic and endoplasmic reticulum (ER) stress pathways (7).

ER is a key organelle of eukaryotic cells, where lipid synthesis, protein folding (into tertiary and quaternary structures) and protein maturation occur. The ER senses and responds to homeostatic changes, with various stimuli, such as ischemia, hypoxia, elevated protein synthesis and Ca^+^ overload-inducing ER stress (8). The ER protein folding capacity is reduced under stress, leading to an accumulation of unfolded proteins. A major response to ER stress is the activation of the glucose-regulated protein 78 (GRP78) through dissociation from its transmembrane receptor, which allows subsequent regulation of the levels of accumulated unfolded proteins (9). Slight and medium ER stress can protect cells from death, but severe ER stress induces caspase-12-dependent cell apoptosis (10).

Numerous compounds have been experimentally shown to ameliorate cisplatin nephrotoxity, including vitamin E, melatonin, furosemide, mannitol and erythropoietin. Grape seed proanthocyanindin extract (GSPE) is derived from grape seeds. It is shown to possess a variety of potent properties, such as antioxidant, anti-inflammatory and antitumor activities, and to mediate resistance to free radicals and protection from cardiovascular diseases (11–13). In this study, GSPE was used to treat the mouse model of CP-induced nephropathy, in order to examine its potential protective effect(s) and examine whether these effects are mediated by the inhibition of ER stress-induced apoptosis occurring in tubular cells.

## Materials and methods

### Reagents

CP was purchased from Sigma-Aldrich (St. Louis, MO, USA). Grape seed proanthocyanidin extract (purity > 96%, lot no. G050412) was purchased from Tianjin Jianfeng Natural Product R&D Co., Ltd. (Tianjin, China). Primary antibodies used in this study were: rabbit anti-GRP78, rabbit anti-caspase-12 (Abcam Ltd., Hong Kong, China), rabbit anti-p-ERK, rabbit anti-ERK (Cell Signaling Technology, Inc., Danvers, MA, USA) and mouse anti-β-actin (Santa Cruz Biotechnology, Inc., Santa Cruz, CA, USA). Anti-mouse and anti-rabbit secondary antibodies were purchased from Jackson ImmunoResearch Laboratories Inc. (West Grove, PA, USA). The In Situ Cell Death Detection kit (Roche Diagnostics, Indianapolis, IN, USA) was used for the terminal deoxynucleotidyl transferase dUTP nick-end labeling (TUNEL) assay.

### Animals

Seventy adult (6–8 weeks-old) male C57/BL6 mice, weighing 20–25 g, were supplied by the Beijing Vital River Laboratory Animal Technology Co., Ltd. (Beijing, China). Mice were housed separately in metal cages with a 12 h dark/light cycle and 40–70% relative humidity, at a 18–22ºC temperature. Food and water were available *ad libitum*. All experiments were conducted in accordance with the NIH Guide for the Care and Use of Laboratory Animals.

### Animal treatment

Animals were randomly divided into four groups: i) control group (N; n=10), which only received intraperitoneal (ip) injection of vehicle solution (0.9% saline; 10 ml/kg); ii) CP group (C; n=20), which only received an ip injection of 20.0 mg/kg CP (dissolved in 0.9% saline to reach a concentration of 2.0 mg/ml); iii) GSPE group (G; n=15), which received a single intragastric (ig) administration of 500 mg/kg GSPE (dissolved in 0.9% saline to reach 50 mg/ml); and iv) CP+GSPE group (C+G; n=20), which successively received ig administration of 500 mg/kg GSPE at 30 min before ip injection of CP, and ig administration of 500 mg/kg GSPE after ip injection of CP 72 h.

The mice were sacrificed 120 h after the injection of 0.9% saline or CP. Prior to sacrifice, the mice were weighed and blood was collected from the endocanthion. From this sample, the serum was separated by centrifugation (912 × g at 4ºC for 20 min) and stored at −80ºC until assayed. Both kidneys were immediately excised and weighed, then each kidney was cut in half by coronal position. Two sections of each excised kidney were stored at −80ºC for western blot analysis. The remaining sections were fixed in 4% buffered paraformaldehyde at 4ºC and embedded in paraffin for histopathologic observation, immunohistochemical study and TUNEL assay.

### Assessments of renal function

Blood urea nitrogen (BUN) and serum creatinine (Scr) levels were measured in a Cobas^®^ 8000 modular analyser (Roche Diagnostics) in Qilu Hospital, Shandong University. The renal index (RI) was calculated as: both kindeys’ weight (g)/animal’s weight (g) ×100.

### Histopathologic observation

The pathologic changes in the kidney were examined by periodic acid-Schiff (PAS) staining. One-fourth of the kidneys was immersion-fixed in 10% buffered formalin and embedded in paraffin to be further examined under a light microscope. Two 4-μm thick sections were performed per animal at an interval of 100 μm and were stained with PAS reagent. Tubular damage was scored as follows: Each section was examined in 5 fields, and the average percentage of the impaired renal tubules was then calculated. The results of renal tubular damage were transformed into an index of renal tubular necrosis, where no damage was assigned a 0 index; <25% damage was assigned 1; 25–50% damage was assigned 2; 50–75% damage was assigned 3 and >75% damage was assigned a 4 index.

### Immunohistochemical study

For immunohistochemical analysis, tissue slices were microwaved for 10–15 min in 0.01% sodium citrate buffer (pH 6.0) to allow antigen retrieval. The tissue slices were cooled at room temperature or in iced water, and then washed with PBS three times. The tissue slices were immersed in 0.1% Triton X-100 for 15 min. To block endogenous peroxidase activity, the tissue slices were incubated with 3% hydrogen peroxide for 10 min in the dark. The tissue slices were then incubated with 10% goat serum for 60 min at 37ºC, and with primary antibody at 4ºC overnight (anti-GRP78 1:200, anti-p-ERK 1:50, anti-caspase-12 1:100), while the sections serving as negative controls were incubated with PBS, instead of the primary antibody. All the sections were incubated with secondary antibodies for 60 min at 37ºC, and stained with 3,3′-diaminobenzidine (DAB) and hematoxylin. Semi-quantitative analysis was performed on the colored sections using a computer imaging analysis system (Leica QWin V3 image analysis software; Leica Microsystems, Heidelberg, Germany). Briefly, 10 high-power fields (×400) per section were randomly selected and two sections per kidney were examined in each experiment. Specimens were scored according to the intensity of the dye color and the percentage of positively stained areas. Brown areas were considered as positive. The intensity of the dye color was graded as 0 (no color); 1 (light yellow); 2 (light brown) and 3 (brown), and the percentage of positively stained areas was graded as 0 (<5%); 1 (5–25%); 2 (25–50%); 3 (51–75%) and 4 (>75%). The two grades were added to give a final score of expression for each tested protein.

### TUNEL assay

The TUNEL assay was conducted following the manufacturer’s instructions. Sections were incubated with proteinase K at 37ºC for 30 min, then with the mix of enzyme and labeling solutions (1:9) at 37ºC for 60 min in the dark. The sections serving as negative controls were incubated with labeling solution only. Sections were then stained with 4′,6-diamidino-2-phenylindole (DAPI) for 10 min. The number of TUNEL-positive nuclei was expressed as a percentage of total nuclei per field. Ten fields per section and two sections per kidney were examined in each experiment.

### Protein sample preparation

The tissue samples were homogenized in TRIzol (50–100 mg/ml TRIzol). Following the addition of chloroform (0.2 ml/ml TRIzol), the homogenates were centrifuged at 12,000 × g for 15 min at 4ºC. Supernatants were discarded, isopropanol (1.5 ml/ml TRIzol) was added to the lower phase, followed by centrifugation at 12,000 × g for 15 min at 4ºC. The pellets were washed with 0.3 M guanidine hydrochloride (2 ml/ml TRIzol) three times, then dissolved in 1% SDS (100 μl SDS/ml TRIzol) at 50ºC for 30 min. Protein concentrations were determined using the Pierce BCA Protein Assay kit (Thermo Fisher Scientific Inc., Rockford, IL, USA).

### Western blotting

Proteins (50 μg) were subjected to 10–12% SDS-polyacrylamide gel electrophoresis, and gels were transferred to cellulose acetate membranes. The membranes were blocked with 5% skim milk at room temperature for 1 h, then incubated with primary antibodies (anti-GRP78 1:250, anti-p-ERK 1:1,000, anti-ERK 1:1,000, anti-caspase-12 1:500 and anti-β-actin 1:2,500) at 4ºC overnight. After a 1-h incubation with secondary antibodies at room temperature, the membranes were immersed in enhanced chemiluminescence (ECL) reagent and exposed to an X-ray film. Quantification of the luminosity of each protein band was performed using Adobe Photoshop software (Adobe Photoshop 7.0, Adobe, San Jose, CA, USA). GRP78, p-ERK and caspase-12 relative quantities were expressed as a ratio of luminosity of the respective sample to that of the N group.

### Statistical analysis

Data are presented as means ± SD. Differences between groups were evaluated using analysis of variance (ANOVA) or Mann-Whitney U-tests. Differences were considered statistically significant at P<0.05.

## Results

### GSPE protects from CP-induced AKI

As shown in Table I, 8 mice out of 10 survived in the N group, 15 mice out of 20 survived in the CP group, 13 out of 15 survived in the GSPE group, and 16 out of 20 survived in the CP+GSPE group. The levels of BUN, Scr and RI were significantly increased in the CP group compared to the control group (P<0.05). Compared to the CP group, the levels of BUN, Scr and RI were significantly decreased in the CP+GSPE group (P<0.05). The levels of BUN, Scr and RI in the GSPE group did not show significant differences compared to the control group.

**Figure 1:**
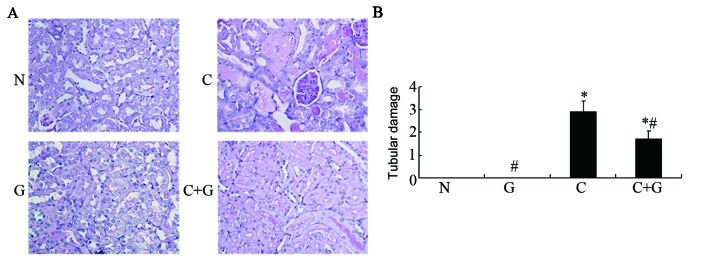
Grape seed proanthocyanidin extract (GSPE) protects from cisplatin (CP)-induced acute kidney injury. Animals were divided into four groups: the control group (N), the CP group (C), the GSPE group (G) and the CP+GSPE group (C+G). (A) Microscopic examination of sections stained with PAS at ×400 magnification. The structure of the kidney was normal in the N and G groups. In the C group, brush border damage, renal tubular epithelial cell swelling, degeneration, necrosis, tubular casts and degeneration of cell vacuoles were observed. The C+G group showed no or very little structural damage. (B) Scores of tubular damage. Data are presented as means ± SD. ^*^Statistically significant difference compared to the N group (P<0.05); ^#^statistically significant difference compared to the C group (P<0.05).

Results from histopathological examinations following PAS staining are shown in Fig. 1A. We observed brush border damage, renal tubular epithelial cell swelling, degeneration, necrosis, tubular casts and cell vacuole degeneration in the proximal tubules of kidney in the CP group, while in the control and GSPE groups, the kidneys maintained normal structure. Compared to the CP group, tubular damage was greatly improved in the CP+GSPE group. We further calculated scores of tubular damage as shown in Fig. 1B. The CP group had an injury score of 2.9, while the CP+GSPE group scored 1.7, and this difference was significant (P<0.05), indicating that GSPE can block the renal injury caused by CP.

### GSPE inhibits apoptosis induced by CP

**Figure 2:**
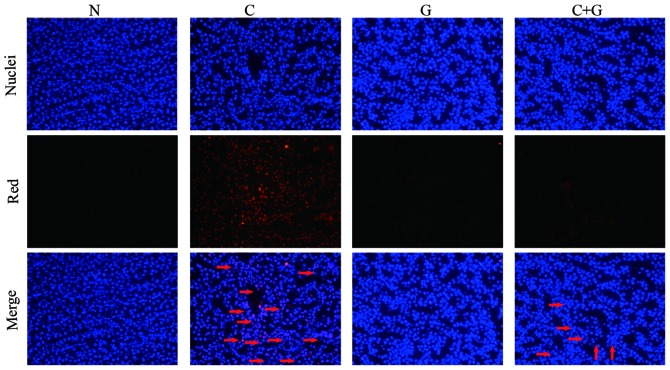
Grape seed proanthocyanidin extract (GSPE) protects from proximal tubular cell apoptosis induced by cisplatin. Arrows on the merge panel show the superposition of nuclear staining to TUNEL (red) staining and represent TUNEL-positive cells. Magnification, ×400. Animals were divided into four groups: the control group (N), the cisplatin (CP) group (C), the GSPE group (G) and the CP+GSPE group (C+G).

**Figure 3:**
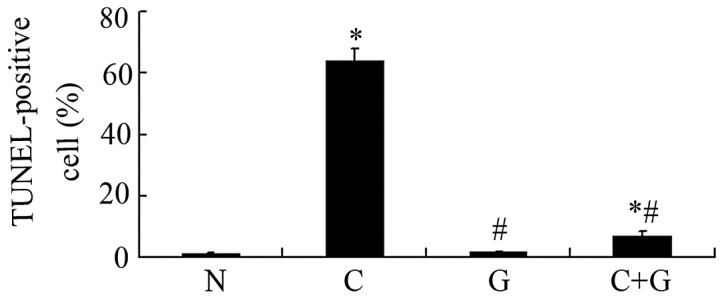
Grape seed proanthocyanidin extract (GSPE) protects from proximal tubular cell apoptosis induced by cisplatin. Animals were divided into four groups: the control group (N), the cisplatin (CP) group (C), the GSPE group (G) and the CP+GSPE group (C+G). ^*^Statistically significant difference compared to the N group (P<0.05); ^#^statistically significant difference compared to the C group (P<0.05).

To assess whether GSPE protects proximal tubular cell apoptosis induced by CP, the tissue sections were labeled with an *in situ* TUNEL assay. As shown in Fig. 2, renal tubular epithelial cells undergoing apoptosis were stained red. The CP group showed a high number of TUNEL-positive cells compared to the control group (P<0.05). The TUNEL-positive cells were significantly reduced in the CP+GSPE group compared to the CP group (P<0.05). Limited apoptosis was detected in the control and the GSPE group (Fig. 3).

### GSPE inhibits expression of GRP78, p-ERK and caspase-12

**Figure 4:**
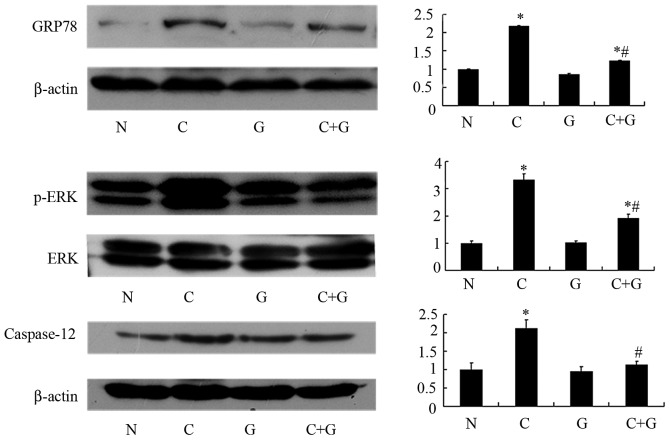
Western blot analysis for GRP78, p-ERK and caspase-12, and quantification of corresponding protein levels. Animals were divided into four groups: the control group (N), the cisplatin (CP) group (C), the grape seed proanthocyanidin extract (GSPE) group (G) and the CP+GSPE group (C+G). Data are expressed as mean ± SD levels relative to β-actin. ^*^Statistically significant difference compared to the N group (P<0.05); ^#^statistically significant difference compared to the C group (P<0.05).

**Figure 5:**
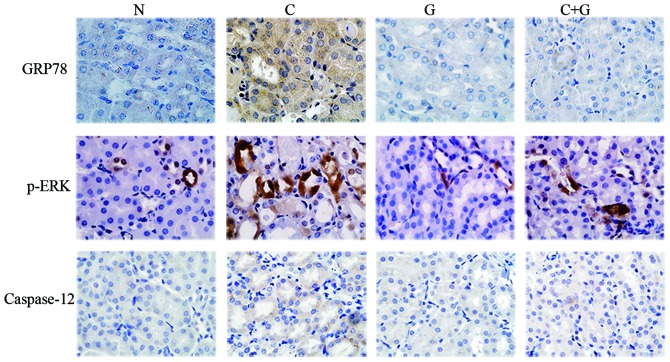
Immunohistochemical staining of GRP78, p-ERK and caspase-12 in the kidney. Animals were divided into four groups: the control group (N), the cisplatin (CP) group (C), the grape seed proanthocyanidin extract (GSPE) group (G) and the CP+GSPE group (C+G). The brown granules represent positively-stained cells. Magnification, ×1,000.

**Figure 6:**
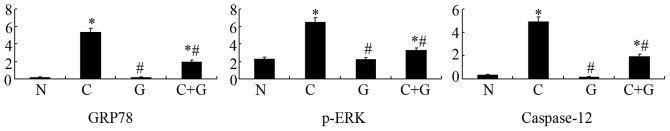
Semi-quantitative analysis of immunohistochemical staining of GRP78, p-ERK and caspase-12 in the kidney, based on the percentage of positively stained areas in the tubules and the intensity of staining. Animals were divided into four groups: the control group (N), the cisplatin (CP) group (C), the grape seed proanthocyanidin extract (GSPE) group (G) and the CP+GSPE group (C+G). The expression of GRP78, p-ERK and caspase-12 was significantly increased in the C group compared to the N group and significantly decreased in the C+G group compared to the C group.^*^Statistically significant difference compared to the N group (p<0.05); ^#^statistically significant difference compared to the C group (p<0.05).

Apoptosis occurred in the proximal tubular cells of the CP group. In order to investigate whether GSPE protects from CP-induced nephrotoxicity by attenuating ER stress-induced apoptosis in proximal tubular cells, we examined the expression of GRP78, p-ERK and caspase-12, which are important protein players in ER stress-induced apoptosis. Expression of these proteins was studied by western blotting and immunohistochemistry. The two approaches showed that GRP78, p-ERK and caspase-12 are highly expressed in the CP group, while their levels are significantly reduced in the CP+GSPE group (P<0.05) (Figs. 4–6). These proteins showed limited expression in the control and GSPE groups.

## Discussion

CP is an antineoplastic drug widely used in the clinic, with obvious curative effects, especially in the treatment of solid-organ tumors; however, nephrotoxicity represents a major dose-limiting side-effect of CP (1). The pathogenesis of CP-induced nephrotoxicity is associated with several factors, including inflammation, oxidative stress, DNA damage, mitochondrial dysfunction and apoptosis (3,6,14,15). Different pathways of apoptosis have been studied in this respect, including the intrinsic and extrinsic pathways (7). It was recently suggested that ER stress-mediated apoptosis is an important pathway in renal apoptosis (16).

In this study, the CP group showed a significant increase in the level of BUN, Cr and RI compared to the control group. Histopathological examination showed that the CP group has significant structural damage compared to the control group. The number of TUNEL-positive cells was significantly increased in the CP group compared to the N group. These results are consistent with those from previous studies (17,18). By contrast, the CP+GSPE group showed a significant decrease in the levels of BUN, Cr and RI compared to the CP group. Histopathology also showed that the CP+GSPE group has very limited structural damage. The number of TUNEL-positive cells was significantly reduced in the CP+GSPE group compared to the CP group. This indicates that GSPE can inhibit the apoptosis of renal tubular epithelial cells induced by CP and may thus improve the renal dysfunction. The mechanism by which GSPE protects from CP-induced nephropathy involves its antioxidant properties (19,20). It has been shown that oxidative stress proteins are downregulated by GSPE in diabetic nephropathy (12,13). GSPE is a highly efficient natural antioxidant extracted from natural grape seeds. Its antioxidant activity is 50 times higher than that of vitamin E and 20 times that of vitamin C. It has also been shown that GSPE has powerful anti-inflammatory effects, which can be used in the treatment and prevention of diseases (21–27). Recent studies provided evidence that GSPE may serve as a useful agent in the prevention of diseases such as atherosclerosis, gastric ulcer, cataract, diabetes, and can also protect from methylmercury-induced neurotoxicity (13,28). In addition, GSPE is known to modulate apoptosis (29). A recent study found that the apoptosis caused by the ER stress pathway plays an important role in CP-induced nephropathy (18). Therefore, CP can lead to apoptosis of the renal tubular epithelial cells by inducing ER stress.

ER stress can be caused by a number of factors such as ischemia, hyperglycemia, hypoxia and heat shock (10). GRP78 is an important molecular chaperone, localized in the ER and extensively used as an indicator of ER stress induction (16). The phosphorylated-extracellular signal-regulated kinase (p-ERK) has an early and crucial role in cell protection and survival during stress, even mild (10). p-ERK is a transmembrane ER protein involved in signal transduction. In the inactive state, ERK and two additional ER-stress sensor proteins, IRE-1 and ATF6, are associated with the ER chaperone Grp78/BiP. When the levels of unfolded and/or misfolded proteins increase, ERK and ER-stress sensors dissociate from Grp78/BiP, and activate downstream molecules (30). The extracellular signal-regulated kinase (ERK) has been shown to mediate CP-induced toxicity in renal proximal tubule cells (31). Caspase-12 plays a key role in ER stress-induced apoptosis (32). Caspase-12 is a marker of apoptosis and specifically localizes in the ER. It was previously demonstrated that caspase-12-mediated apoptosis is specific to ER, and that caspase-12 cannot be activated when apoptosis occurs via membrane or mitochondrial signals (33). Thus, GRP78 and p-ERK are protein markers of ER stress and caspase-12 is a marker of ER stress-induced apoptosis.

To determine the mechanism by which GSPE protects tubular cells from CP-induced apoptosis, the protein levels of GRP78, p-ERK and caspase-12 were measured. GRP78, p-ERK and caspase-12 were highly expressed in CP-treated mice that showed important structural alterations in the kidney, indicating that CP-induced nephropathy involves ER stress-induced apoptosis. GSPE can attenuate ER stress-induced apoptosis, as evidenced in the present study by the significant reduction in the levels of GRP78, p-ERK and caspase-12, observed following administration of the extract. The present results suggest that exposure to CP activates ER stress-regulated survival and apoptotic signaling pathways in renal tubular cells. Moreover, we present evidence that the caspase-12-dependent apoptotic pathway may be involved in CP-induced nephropathy. The reduction in the expression of GRP78, p-ERK and caspase-12 caused by GSPE demonstrates that GSPE can attenuate ER stress-induced apoptosis via the caspase-12-dependent pathway.

In conclusion, this study showed that GSPE can protect from CP-induced AKI. The underlying mechanism involves the inhibition of ER stress-mediated apoptosis via the caspase-12-dependent pathway. These results suggest that GSPE can be applied to treat CP-induced nephropathy.

## Figures and Tables

**Table I:** Animal groups and related biochemical parameters.

Group	No.	RI	BUN	Scr
N	8	1.43±0.074	10.19±1.17	29.5±6.07
C	15	1.79±0.066^a^	30.85±6.74^a^	135.13±12.64^a^
G	13	1.41±0.068^b^	10.23±1.44^b^	33.08±5.88^b^
C+G	16	1.64±0.039^a^,^b^	14.26±2.66^a^,^b^	62.81±9.55^a^,^b^

Data are presented as means ± SD, No., number of mice in the group; RI, renal index; BUN, blood urea nitrogen level; Scr, serum creatinine level; N, control group; C, cisplatin (CP) group; G, grape seed proanthocyanidin extract (GSPE) group; C+G, CP+GSPE group;

a statistically significant difference compared to the N group (P<0.05);

b statistically significant difference compared to the C group (P<0.05).

## Abbreviations

CP: cisplatin
GSPE: grape seed proanthocyanidin extract
ER: endoplasmic reticulum
GRP78: glucose-regulated protein 78
BUN: blood urea nitrogen
Scr: serum creatinine
RI: renal index
AKI: acute kidney injury
